# Supplementary material for: Causal Relationship between Aging and Anorexia Nervosa: A White-Matter-Microstructure-Mediated Mendelian Randomization Analysis
Source: Biomedicines. 2024 Aug 16;12(8):1874. doi: 10.3390/biomedicines12081874 (PMC11351342; doi:10.3390/biomedicines12081874)
Supplement: Supplementary file 1 [file biomedicines-12-01874-s001.zip › Supplementary Figure/Figure S8/Leaveoneout plot of MR analysis for TL to ubm-b-2002.pdf]

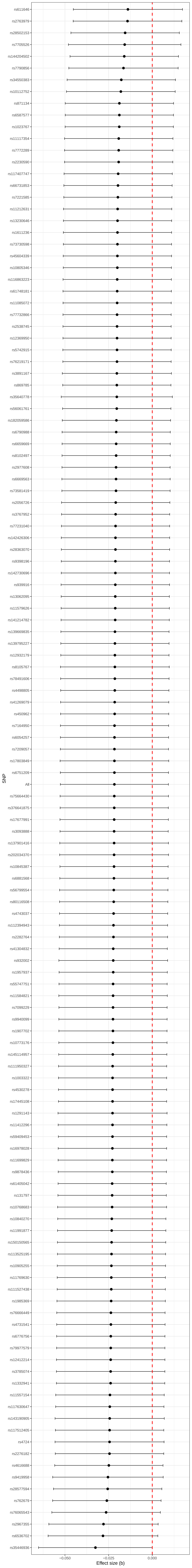

rs611646
rs2763979
rs28502153
rs7705526
rs144204502
rs7790856
rs34550383
rs10112752
rs871134
rs6587577
rs1023767
rs11117354
rs7772289
rs2230590
rs117407747
rs66731853
rs7221585
rs11212631
rs13230646
rs1611236
rs73730598
rs45604339
rs10805346
rs116863223
rs61748181
rs11085072
rs77732866
rs2538745
rs12369950
rs5742915
rs76219171
rs3891167
rs869785
rs35640778
rs56061761
rs182059586
rs6790988
rs6659669
rs8102497
rs2977608
rs6669563
rs73581419
rs2056726
rs3767952
rs77231040
rs142426306
rs28363070
rs9398196
rs142730696
rs939916
rs13062095
rs11579626
rs141214782
rs139669835
rs139795227
rs12932179
rs8105767
rs78491606
rs4498805
rs41269079
rs450962
rs7164950
rs6054257
rs7209057
rs17803849
rs6751209
All
rs75664430
rs376641875
rs17677991
rs3093888
rs137901416
rs202034370
rs10845387
rs6881568
rs56799554
rs80116508
rs4743037
rs112394943
rs2282764
rs41304832
rs932002
rs1957937
rs55747751
rs11584821
rs7099229
rs9940099
rs1907702
rs10773176
rs145114957
rs111950327
rs1003322
rs4530278
rs17445108
rs1291143
rs11412296
rs59409453
rs16978028
rs11699829
rs9878436
rs61405042
rs131797
rs10768683
rs10840270
rs11991877
rs150150565
rs10905255
rs113525195
rs11769630
rs111527438
rs1985369
rs76666449
rs4731541
rs6776756
rs79977579
rs12412214
rs3785074
rs1332941
rs11557154
rs117630647
rs143190905
rs117512405
rs4724
rs2276182
rs4616688
rs9419958
rs28577594
rs762679
rs76065543
rs2967355
rs6536702
rs35446936
SNP
-0.050
-0.025
0.000
Effect size (b)
